# Fifth Distal Phalanx Avulsion Fracture in a High-Level Marathon Runner

**DOI:** 10.7759/cureus.37468

**Published:** 2023-04-12

**Authors:** Eric Chun-Pu Chu, Steve Ming Hei Yun, Kevin Hsu Kai Huang

**Affiliations:** 1 New York Chiropractic and Physiotherapy Centre, New York Medical Group, Hong Kong, HKG; 2 New York Chiropractic and Physiotherapy Centre, New York Medical Group, Kowloon, HKG

**Keywords:** metatarsal fractures, phalanx, avulsion fracture, chiropractor, chiropractic

## Abstract

Fifth metatarsal fractures are common foot injuries that involve the long bone on the outer side of the foot, and avulsion fractures involving the short bone and the fifth distal phalanx of the foot have never been reported. A 25-year-old female marathon runner sustained an avulsion fracture of the distal lateral phalanges of the fifth metatarsal. The patient's high functional demands necessitated a conservative approach to minimize complications and facilitate efficient fracture healing. The patient underwent a comprehensive chiropractic rehabilitation program that focused on progressive weight-bearing exercises, range-of-motion activities, strengthening exercises, instrument-assisted soft tissue mobilization (IASTM), therapeutic ultrasound, and laser therapy to stimulate the speed of healing. The patient's progression was closely monitored throughout the rehabilitation process. Because of the nonoperative management and chiropractic rehabilitation, the patient successfully returned to her running activities within a six-week duration. This case demonstrates the effectiveness of nonoperative management and chiropractic rehabilitation in promoting the healing of avulsion fractures of the fifth metatarsal in high-level athletes. This conservative approach can facilitate a safe and efficient return to running activities while minimizing complications and reinjury risks.

## Introduction

The most common foot and ankle injuries are metatarsal fractures, which occur 6.7 times per 100,000 people each year [1]. The fifth metatarsal bone base is involved in 30% of metatarsal fractures due to the anatomy and biomechanics of the pes quintus (fifth foot) [1]. The tuberositas ossis metatarsi quinti, which is a projection on the lateral surface of the fifth metatarsal bone and serves as an insertion point for ligaments and tendons [2], is highly prominent and increases the lever arm of the bone, leading to greater stress in this area [3]. Avulsion fractures of the tuberosity are frequent and caused by the sudden inversion and plantar flexion of the foot [4]. The diaphysis of the fifth metatarsal bone is also susceptible to fractures due to rotational and torsional forces applied to the forefoot during locomotion or trauma [5]. The osseous anatomy and forces applied to the lateral column of the foot predispose the fifth ray to injuries [6]. Proper diagnosis and treatment are crucial for addressing these fractures and promoting a successful recovery [7].

Chiropractic is a separate form of healthcare that places special emphasis on the spinal balance and nervous system as well as the overall structure and function of the body [8]. In Hong Kong, chiropractors often manage neuro-musculoskeletal disorders [9], and they frequently manage high-performance sports athletes [10-12]. We present a rare sports injury caused by an avulsion fracture involving the distal phalanx of the foot. This case is unique as most avulsion fractures typically occur at the proximal phalanx or base of the metatarsal. We searched the medical literature using PubMed, Google Scholar, and Scopus databases but found no previously reported cases of avulsion fractures situated at the distal phalanx. Given the atypical nature of this case study, it is imperative to highlight the successful nonoperative management of a fifth distal lateral phalanx injury in a marathon runner that allowed for a return to running activities.

## Case presentation

A 25-year-old female marathon runner presented to the chiropractic clinic with sudden onset right fifth metatarsal pain four weeks prior to her marathon training. Her training regimen typically includes a mix of long-distance runs (between 15 and 22 km), threshold runs, and interval workouts, structured over several months to optimize performance. Alongside these core running workouts, she also incorporates strength training, flexibility exercises, and cross-training activities such as cycling or swimming to build overall strength, reduce the risk of injury, and improve her performance on race day. She noticed a bruise that developed over the right fifth metatarsal after completing the run one month prior. She had been applying ice to the area for 48 hours, and the bruise slowly recovered. However, the pain was a constant dull ache, rated 4 out of 10 on the numeric pain scale over the past four weeks. No associated numbness, tingling, or skin changes were observed. She was unable to wear narrow or high-heeled shoes. She had no history of foot pain or injury, and she had no significant past medical or surgical history. However, she reports experiencing recurrent ankle sprains during the past five years, often occurring during training sessions or competitive events.

**Figure 1:**
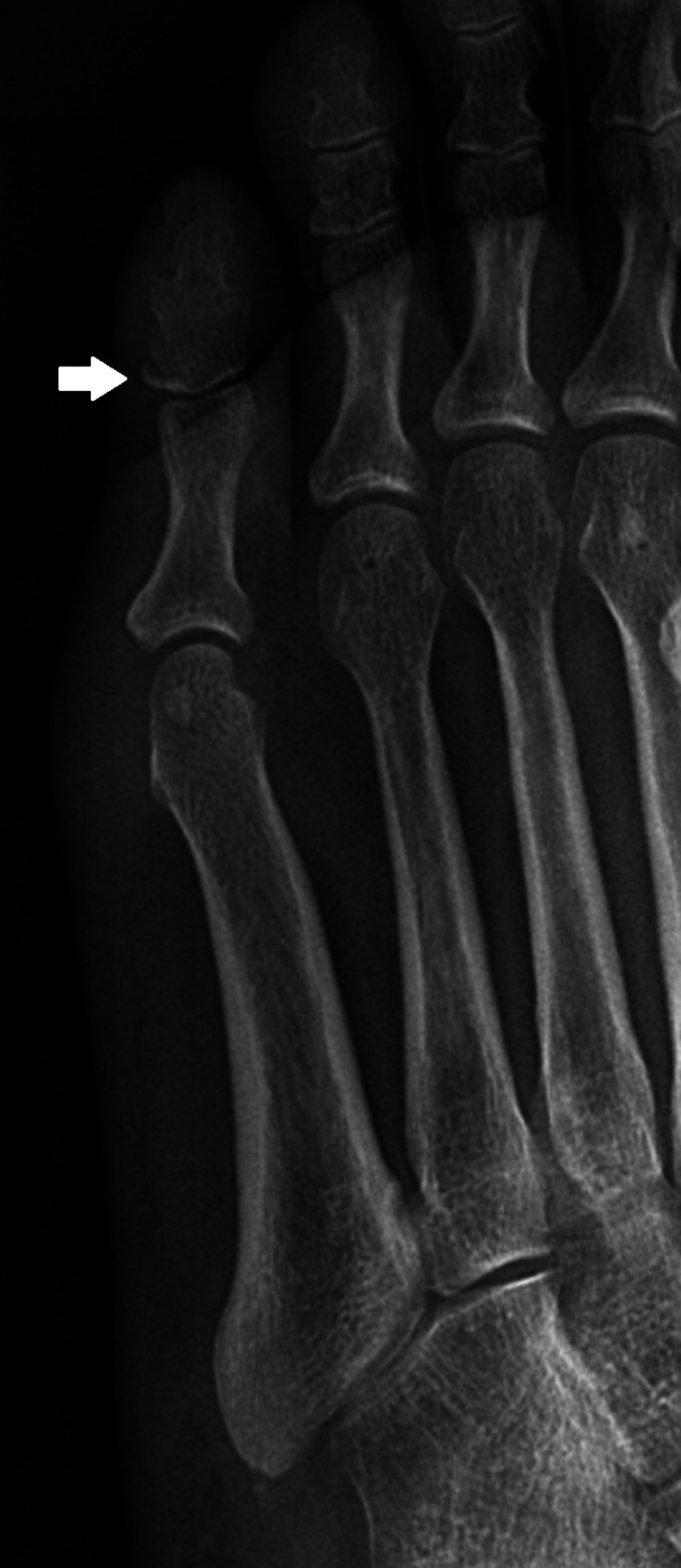
Radiograph of the right foot A non-displaced avulsion fracture of the superolateral aspect of the right distal fifth phalanx

The patient presented with vital signs at the chiropractic clinic. The pulse, capillary refill, and skin temperature of the right foot were normal. Deep tendon reflexes in the ankle and knee were normal bilaterally. Sensory examination revealed normal light touch sensation over the right fifth metatarsal region. The vibration sensation was intact bilaterally at the great toe. However, dorsiflexion of the lateral four toes against resistance revealed a minor weakness graded as 4/5. Palpation revealed swelling at the right fifth metatarsal, as well as an overactive and tight extensor digitorum longus and tibialis anterior. There were palpable tender nodules located on the dorsal surface of the plantar area, and the trigger points were hyperalgesic when touched. The nodules compressed the extensors of the ankle. Radiography of the right foot revealed a non-displaced avulsion fracture of the superolateral aspect of the right distal fifth phalanx (Figure 1). No other fractures or dislocations were observed. Based on the anatomical structure, clinical evaluation, and radiological findings, the diagnosis was an avulsion fracture of the extensor digitorum longus at the distal phalanx. The patient was arranged to consult with an orthopedic surgeon in the same clinic and was recommended for chiropractic care.

The patient’s prognosis for recovery is guarded due to her high activity level as a marathon runner. The initial treatment plan included complete avoidance of weight-bearing and the use of an offset-loading brace for four weeks, as well as spinal manipulation of the lumbar and pelvic joints to balance biomechanics. Once the pain improved, an intensive program involving range of motion and strengthening exercises was added and tailor-made for rehabilitation. Stretching and instrument-assisted soft tissue mobilization (IASTM) of the muscle, together with activation of the tibialis anterior, are indicated to regain muscle balance and improve functional ankle dorsiflexion. Low-intensity pulsed ultrasound and laser therapy were administered to facilitate healing, and manual therapy was performed on the proximal foot joints three times a week to increase mobilization and function. In week 3, a re-evaluation showed a reduction in pain score to 2/10 and the patient’s gait returned to normal. The patient’s scheduled frequency of chiropractic care was reduced to twice a week with close monitoring of the avulsion fracture to prevent a complete fracture in this highly active patient. After rest and chiropractic care, the patient was able to return to running activities within six weeks. During the three-month follow-up visit, all pain was resolved, and dorsiflexion of the lateral four toes against resistance revealed normal. She had recovered fully without complications.

## Discussion

Avulsion fractures of the fifth toe involve multiple musculoskeletal structures. The peroneus brevis tendon inserts into the tuberosity ossis metatarsi quinti and acts to evert and plantar flex the foot. [13]. A sudden inversion strain can cause this tendinous insertion to fracture the eminentia [14]. The plantar fascia and flexor digitorum brevis are also attached to the base of the fifth metatarsal, stabilizing the bone through plantarflexion [2]. The calcaneofibular ligament supports the lateral column of the foot and provides lateral stability to the talocrural joint, while the anterior talofibular ligament resists excessive supination [3]. Tension in these ligaments from excessive inversion can induce the avulsion of the attached tendons. The peroneus tertius also inserts into the dorsal surface of the fifth metatarsal base, providing dorsiflexion and eversion [15]. The distal metatarsal phalange is attached to the extensor digitorum longus, which extends the toes and contributes to ankle dorsiflexion. During injury, the extensor digitorum longus can create significant force, leading to avulsion fractures of the tuberosity. However, acute ankle sprains that lead to extensor digitorum longus and fascia injuries are rare [16].

Although the scientific evidence does not support a standard treatment for fifth toe fractures [1], the choice of treatment generally depends on the fracture's location and displacement. Non-operative management is often the preferred initial treatment for avulsion and non-displaced diaphyseal fractures. This may include immobilization with a short leg cast, walking boot, or rigid shoe, along with limited weight-bearing for a period of four to six weeks [17]. Following this period, progressive weight-bearing and physical therapy can be initiated to restore function and strength. Nonoperative treatment has demonstrated favorable outcomes in patients with stable fractures and minimal displacement [18]. Surgical intervention is generally reserved for fractures with significant displacement, comminution, or those at high risk of nonunion due to the limited blood supply at the metaphyseal-diaphyseal junction and may necessitate surgical fixation with intramedullary screw fixation or tension band wiring [17]. Displaced diaphyseal fractures may also require open reduction and internal fixation with plates and screws for adequate stability [19]. Surgical treatment has been shown to expediate healing and reduce the risk of complications, particularly in patients with a high likelihood of nonunions [20].

In this case, the non-operative treatment approach is well justified, given the non-displaced nature of the avulsion fracture and the high functional demands of the patient. Nondisplaced fractures inherently maintain alignment and stability, allowing efficient bone healing without the need for surgical intervention [21]. Furthermore, although the distal metatarsal phalanx is not the main weight-bearing joint, bracing, immobilization, and rest for four weeks may provide adequate support and protection for the healing fracture [17]. After the initial immobilization and limited weight-bearing phases, a structured rehabilitation program should be implemented that focuses on progressive weight-bearing, range-of-motion exercises, and strengthening [16]. Our conservative approach, including the use of rehabilitative exercises, IASTM, ultrasound, laser therapy, and manual therapy to the proximal joints, also plays a pivotal role in the nonoperative management of fifth metatarsal fractures, ensuring a successful return to activities and minimizing the risk of reinjury. Gradual reintroduction of weight-bearing activities enhances the healing process while mitigating the risk of delayed union or nonunion [17]. This conservative treatment modality minimizes the risk of complications associated with surgery such as infection, nerve or vascular injury, and hardware-related issues [22].

This case report provides preliminary evidence for the non-operative chiropractic management of a fifth metatarsal avulsion fracture. As avulsion fractures at the distal phalanx are rare, the findings of this single case may not be broadly applicable to other patients. However, the non-operative treatment strategy offers a safe and efficient means to address non-displaced fractures while accommodating the patient's high functional demands.

## Conclusions

In conclusion, this case involved a nondisplaced fifth metatarsal fracture in a patient with high functional demands. After the orthopedic surgeon decided it was a non-surgical case, rehabilitative management consisting of immobilization and a structured rehabilitation program was successfully implemented to address the injury. Chiropractic care includes range-of-motion exercises, strengthening, IASTM, therapeutic ultrasound, laser therapy, and manual therapy of the proximal joint tailored to the patient's specific needs and goals. A comprehensive and individualized approach to nonoperative management facilitated a favorable outcome for the patient, enabling a safe and efficient return to running. This case highlights the efficacy of conservative chiropractic management in promoting the healing of non-displaced fifth metatarsal fractures at the distal metatarsal phalanx. No complications or issues with the conservative approach were reported, supporting the fact that it was a safe method of treatment for this patient. The conservative approach also facilitates high-level athletes to resume their activities in a relatively short period of time.
